# Feasibility of neoadjuvant immunochemotherapy in potentially resectable non-small cell lung cancer: a single-arm meta-analysis

**DOI:** 10.3389/fonc.2026.1826159

**Published:** 2026-05-07

**Authors:** Fengpin Wu, Jia Duan, Zongmin Shi, Yuanmei Zhang, Lei Yang, Duo Li

**Affiliations:** 1 Department of Geriatrics, Neijiang First People’s Hospital, Neijiang, Sichuan, China; 2 Department of Intensive Care Medicine, Neijiang Hospital of Traditional Chinese Medicine, Neijiang, Sichuan, China; 3 Department of Hospital Infection Management, Affiliated Hospital of Southwest Medical University, Luzhou, Sichuan, China; 4 Department of Pulmonary and critical care medicine, Affiliated Hospital of Southwest Medical University, Luzhou, Sichuan, China

**Keywords:** meta-analysis, neoadjuvant immunochemotherapy (NICT), NSCLC, potentially resectable, single-arm

## Abstract

**Background:**

Potentially resectable, stage IIB–IIIB non-small cell lung cancer (NSCLC) presents a significant therapeutic challenge. Factors such as tumor size, location, or nodal status often preclude initial complete resection, necessitating novel strategies to improve prognosis. While neoadjuvant immunochemotherapy has demonstrated promise in metastatic/advanced NSCLC, evidence for its application in the potentially resectable setting remains limited. This study synthesizes the existing evidence and evaluates the efficacy, pathological response rates, and frequency of treatment-related events associated with neoadjuvant immunochemotherapy in this specific patient population.

**Methods:**

A single-arm meta-analysis was conducted following the PRISMA (Preferred Reporting Items for Systematic Reviews and Meta-Analyses)-NMA guidelines. A systematic search of databases (PubMed, Embase, and Cochrane) was performed up to 9 December 2025, for studies investigating neoadjuvant anti-PD-1/PD-L1 therapy combined with chemotherapy in potentially resectable NSCLC. Data were analyzed using STATA 14. A random-effects model was employed for significant heterogeneity (*I*^2^ > 50%); otherwise, a fixed-effects model was used. Sensitivity analyses, Begg’s test/Egger’s test, and the trim-and-fill method were applied to assess bias.

**Results:**

A total of 34 studies involving 1,885 patients were included. The pooled surgical resection rate was 76.0% (95% CI: 70.0%−82.0%; *I*^2^ = 89.34%). The R0 resection rate was 100% (95% CI: 99%−100%; *I*^2^ = 0.00%). The pathological complete response (pCR) rate was 35.9% (95% CI: 30.7%−41.0%; *I*^2^ = 13.94%), and the major pathological response (mPR) rate was 25% (95% CI: 21%−29%). The combined rate of pCR and mPR was 60.2% (95% CI: 55.1%−65.4%; *I*^2^ = 65.70%). The incidence of grade ≥3 adverse events (AEs) was 9.0% (95% CI: 5.0%−14.0%; *I*^2^ = 82.05%), and the rate of surgical complications was 8.0% (95% CI: 3.0%−15.0%; *I*^2^ = 82.37%). Sensitivity analyses and the trim-and-fill method supported the robustness of the results, with no significant publication bias detected.

**Conclusion:**

This single-arm meta-analysis suggests that for patients with potentially resectable NSCLC, neoadjuvant immunochemotherapy followed by surgery shows promising short-term pathological response rates and acceptable rates of treatment-related events, indicating potential feasibility. However, because of the lack of comparator data and mature long-term survival outcomes, its definitive clinical benefit remains uncertain. Our results provide preliminary evidence and rationale for initiating randomized controlled clinical trials in this population to compare the efficacy and safety of surgery after neoadjuvant immunochemotherapy versus current standard therapy.

**Systematic Review Registration:**

https://www.crd.york.ac.uk/prospero/, identifier CRD42024579329.

## Introduction

Lung cancer is one of the leading causes of cancer incidence and mortality worldwide (1). Patients with locally advanced stages show high heterogeneity, often necessitating multidisciplinary discussion. The current standard treatment for unresectable locally advanced non-small cell lung cancer (NSCLC) is concurrent chemoradiotherapy followed by durvalumab maintenance therapy (PACIFIC model) (2). However, survival outcomes remain suboptimal compared to patients with surgically resectable NSCLC, highlighting the urgent need for new approaches to improve the prognosis of potentially resectable NSCLC.

Adding immunotherapy to standard chemotherapy, as opposed to chemotherapy alone, as first-line treatment for metastatic or advanced NSCLC significantly improves progression-free survival (PFS) and overall survival (OS) (3–5). With the advancement of immunotherapy from the late to the perioperative stage, multiple studies have indicated that immunotherapy combined with or without chemotherapy can achieve major pathological responses (mPRs) in most patients, leading to high R0 resection rates and controllable treatment-related toxicity (6–10). Radical surgery following chemotherapy appears to be safe and effective in patients with unresectable locally advanced NSCLC.

Potentially resectable NSCLC refers to locally advanced NSCLC (clinical stage IIB–IIIC) that is deemed technically resectable by a multidisciplinary team (MDT) assessment, but poses challenges for initial complete resection due to factors such as tumor size, location, and lymph node status (primarily N2, with some cases involving T3–4 or N1), resulting in high surgical risk or an anticipated poor prognosis. It remains relatively unknown whether neoadjuvant immunochemotherapy can convert initially unresectable NSCLC to resectable NSCLC and whether subsequent tumor resection can improve long-term survival.

Many studies have explored neoadjuvant immunotherapy combined with chemotherapy; however, small sample sizes and inconsistent findings have prevented a consensus on the feasibility of neoadjuvant immunochemotherapy in potentially resectable NSCLC, highlighting the lack of robust clinical data. Thus, systematic reviews and meta-analyses are urgently needed to assess the feasibility of neoadjuvant immunotherapy for potentially resectable NSCLC to guide clinical practice. We hope that the findings of this study will synthesize the existing evidence and provide a theoretical basis for the design of future clinical trials.

## Study design

This systematic review and meta-analysis adhered to the PRISMA (Preferred Reporting Items for Systematic Reviews and Meta-Analyses)-NMA reporting guidelines. The study protocol was registered with PROSPERO (CRD42024579329).

### Search strategy and inclusion criteria

We included clinical studies published from the inception of the PubMed, Embase, and Cochrane databases to the present (last search update: 9 December 2025). These studies pertained to neoadjuvant immunochemotherapy for potentially resectable lung cancer, with language restriction to English. The full search strategy is presented in **Supplementary Table S1**.

We included published studies on neoadjuvant immunochemotherapy, including prospective, observational, and retrospective studies, as well as conference abstracts (if containing complete data). We acknowledge that including conference abstracts may introduce bias due to incomplete data or lack of peer review; therefore, a sensitivity analysis excluding conference abstracts was planned to assess their impact on the overall results (see the Results section). Editorials, reviews, gray literature, duplicate publications, systematic reviews, trial designs, case reports with a sample size smaller than 10, studies that did not report relevant outcomes, or studies in which relevant data could not be retrieved were excluded. Additionally, studies involving patients receiving radiotherapy, anti-angiogenic drugs, and neoadjuvants; those with resectable NSCLC; or stage IV patients were excluded. Studies involving only adult patients were included.

All articles were screened by title and abstract by two independent reviewers (WFP and DJ), and the results were consolidated by YL. Disagreements were resolved through discussion. Outcomes included interventions with neoadjuvant treatment using anti-PD-1/PD-L1 combined with chemotherapy. Outcome measures included pathological complete response (pCR) and mPR; surgical indicators included surgery rate and R0 resection rate; and safety indicators included grade 3–5 adverse events (AEs) and perioperative complication incidence, among others. It should be noted that our assessment of “surgical safety” is limited to the reported incidence of postoperative complications. Key intraoperative metrics reflecting surgical complexity and feasibility, such as conversion rates from minimally invasive to open surgery, operative time, estimated blood loss, and extent of lung resection, were not consistently reported and thus could not be meta-analyzed. For unreported key variables (e.g., age and sex), complete case analysis was applied. Two independent reviewers (WFP and DJ) screened studies for inclusion based on predefined criteria. Discrepancies were resolved through discussion. Cohen’s kappa coefficient was calculated to assess inter-rater agreement, yielding a moderate level of consistency (κ = 0.49, 80% overall agreement).

#### Definition of potential resectability

In this review, we operationally defined “potentially resectable NSCLC” as including T4 invading the esophagus, heart, aorta, or pulmonary veins; N2 patients with single-station mediastinal lymph node short axis ≥3 cm or multi-station confluent lymph nodes (short axis ≥2 cm on CT); and all N3 patients. This group included patients who, after MDT assessment, were deemed to have a low likelihood or high risk for direct surgical resection, but for whom it was still considered technically feasible following significant tumor volume reduction with systemic therapy. It is important to note that the specific definition of “potentially resectable” likely varied across the included studies based on their respective institutional MDT criteria, which is a significant source of clinical heterogeneity in this analysis.

### Quality assessment

Quality was evaluated by two independent reviewers (WFP and DJ) using the JBI Critical Appraisal Checklist for Case Series (https://jbi.global/critical-appraisal-tools), and any discrepancies were resolved by a third reviewer (SZM). The risk of bias in the included studies was assessed according to a checklist in which each of the 10 methodological questions had to be answered with one of two options: yes (Y) and no (N). Studies were considered eligible for inclusion if they had more than four Y’s and sufficient outcome indicators. The risk of bias was quantified based on the number of Y answers and was categorized as high (≤49%), medium (50%–70%), and low (≥71%). This report complies with the PRISMA extension guidelines for systematic reviews that include single-arm meta-analyses of healthcare interventions.

### Statistical analysis

All data for this meta-analysis were analyzed using STATA 14 software. Heterogeneity was assessed using the χ² test (*p* < 0.1 indicating significant heterogeneity) and *I*^2^ statistic (*I*^2^ > 50% denotes moderate-to-high heterogeneity). When significant heterogeneity was detected (*p* < 0.1, *I*^2^ > 50%), a random-effects model was applied. Otherwise, a fixed-effects model was used (11). Sensitivity analysis was also conducted to assess the stability and reliability of the pooled results. Finally, potential publication bias was detected using the Begg’s and Egger’s tests.

To assess potential publication bias, the trim-and-fill method was applied using a linear estimator under fixed-effects model or random-effects model. This nonparametric approach iteratively estimates the number of missing studies due to funnel plot asymmetry and imputes hypothetical studies to adjust the pooled effect size. Analyses were conducted in STATA 14 (metatrim command) with a maximum of 1,000 iterations.

## Results

**Figure 1:**
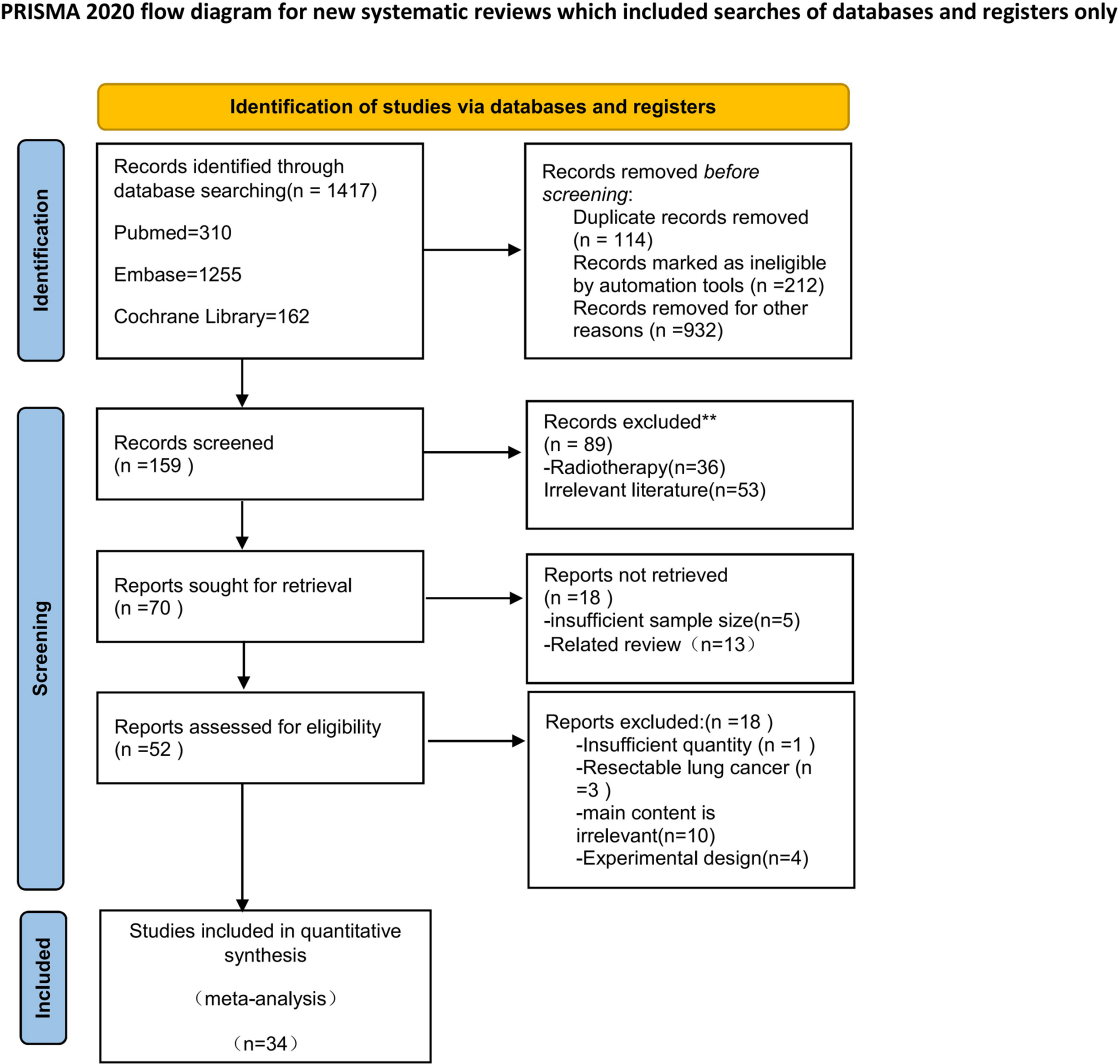
Flow diagram. *Consider, if feasible to do so, reporting the number of records identified from each database or register searched (rather than the total number across all databases/registers). **If automation tools were used, indicate how many records were excluded by automation tools. Source: Page MJ, et. al. BMJ 2021;372:n71. doi: 10.1136/bmj.n71. This work is licensed under CC BY 4.0. To view a copy of license, visit https://creativecommons.org/licenses/by/4.0/.

The initial search yielded 1,417 articles from three databases (PubMed = 310, Embase = 1,255, Cochrane Library = 162). Approximately 52 studies were retained after duplicates were removed, and titles and abstracts were screened. A total of 37 studies were included in full-text screening. Subsequently, it was identified that three of these studies were descriptions of results from different periods of the same study; therefore, the conclusions from the latest one were used. The selection process is depicted in **Figure 1**.

The 34 included studies (12–47) comprised a total of 1,885 patients. Among them, 17 studies (50%) were prospective in design, while 17 (50%) were retrospective. The sample sizes of individual studies ranged from 10 to 469 patients. The most commonly used immune checkpoint inhibitors (ICIs) were tislelizumab (*n* = 9 studies), toripalimab (*n* = 4 studies), sintilimab (*n* = 4 studies), and pembrolizumab (*n* = 1 study); the remaining studies (*n* = 18) employed various other ICIs or combinations, reported collectively as “ICI”. The median or mean age of patients across studies ranged from 58 to 68 years. The majority of patients were male (reported range: 54.3% to 100% across studies where data were available) and had a history of smoking. Pathologically, squamous cell carcinoma was the predominant subtype in most studies. All patients were diagnosed with locally advanced (stage IIB, IIIA, IIIB, or IIIC) NSCLC, with staging predominantly based on the AJCC 8th edition (JACC = 8). The neoadjuvant treatment regimen typically consisted of two to four cycles of ICI combined with platinum-doublet chemotherapy, and the details of each included study are presented in **Table 1**.

**Table 1:** Characteristics of the studies included in the meta-analysis.

Study	Year	Article	Type	Sample	Interventions	Age	Male	Female	Smoking	Squamous	Non-squamous	Cycle	IIA	IIB	IIIA	IIIB	IIIC	JACC
S. Yan	2021	Meeting	Prospective	21	Toripalimab	62 (45–74)	19	2	18	16	5	2–4		6	11	4		NA
T. Wang	2022	Meeting	Prospective	33	Tislelizumab	NA	NA	NA	NA	NA	NA	2			13	5		NA
Y. Zhang	2021	Meeting	Prospective	56	ICI	58 (46–74)	54	2	NA	47	9	2–4			41	15		NA
X. Zhu	2021	Article	Prospective	48	Toripalimab	66 (34–77)	41	7	34	31	17	2		4	24	15	5	NA
Hao Chen	2024	Article	Retrospective	12	Tislelizumab	NA	10	2	12	12	0	3	4	1		7		8
Jing Zheng	2023	Article	Retrospective	56	Pembrolizumab	64.8 ± 8.1	56	3	45	43	13	2–4				56		NA
Hongyan Xu	2022	Meeting	Retrospective	14	ICI	68	14	0	14	14	0	2–4			8	6		8
Chao Sun	2024	Article	Prospective	30	Sintilimab	60.5 (53.5–64.0)	29	1	28	26	4	2–3			20	10		8
Y. Zhang	2022	Meeting	Prospective	33	Toripalimab	58	29	4	27	29	4	2			17	15		NA
Y. Wang	2023	Article	Prospective	24	Tislelizumab	NA	19	5	15	16	8	2–4		4	10	9	1	NA
Haoran Xia	2024	Article	Prospective	30	Camrelizumab	65 (19–74)	28	2	25	20	10	2–4		1	26	3		8
X. Dong	2022	Article	Prospective	14	Durvalumab	64.5	NA	NA	NA	10	4	3–4			2	10	2	NA
Jing Guo	2023	Meeting	Retrospective	17	ICI	64.8 ± 7.7	17	0	12	17	0	2–4			10	7		8
Tianxiang Chen	2021	Meeting	Retrospective	12	ICI	61 (55.3–66.8)	9	2	9	6	6	2–4			7	5		8
Jianzhen Shan	2024	Article	Prospective	35	Tislelizumab	65 (48–70)	35	0	34	30	0	4			30	5		8
G. Jiang	2023	Article	Prospective	30	Camrelizumab	NA	NA	NA	NA	NA	NA	2–4						NA
K. Ma	2023	Meeting	Prospective	30	Sintilimab	NA	NA	NA	NA	NA	NA	2–4						NA
Hongsheng Deng	2021	Article	Retrospective	51	ICI	62 (56–67)	44	7	40	34	17	4				51		8
Ying Wang	2023	Article	Retrospective	65	ICI	67 (62–71)	65	0	49	65	0	2–4			38	27		8
J.Z. Shan	2022	Meeting	Prospective	35	Tislelizumab	65 (48–78)	35	0	NA	35	0	2–4			29	6		8
Xuhua Huang	2024	Article	Retrospective	39	Tislelizumab	68.5 ± 6.63	37	2	25	34	5	2–4		6	33			8
Min Tang	2025	Article	Prospective	59	Tislelizumab	65 (46–82)	54	5	NA	40	19	3–4			20	27	12	8
T. Wang	2024	Article	Retrospective	75	Tislelizumab	57.0 (46.0–73.0)	74	1	NA	NA	NA	2–4				75		NA
Biagio Ricciuti	2025	Article	Retrospective	112	ICI	66 (41–84)	54	58	99	NA	76	NA			70	27	6	8
Suyu Wang	2024	Article	Retrospective	469	ICI	NA	255	23	187	NA	NA	1–4			94	169	15	8
Xiangyang Yu	2024	Meeting	Prospective	30	Sintilimab	64.4 ± 6.2	23	7	18	8	19	2				30		8
Jianfeng Li	2024	Article	Retrospective	46	ICI	58.5 (52.0–64.0)	38	8	18	39	7	1–4			33	13		8
Yana Qi	2025	Meeting	Retrospective	195	ICI	62.58 ± 7.59	175	66	204	145	50	2			212	144	51	8
Chengxiang Yi	2025	Meeting	Prospective	79	Sintilimab	63.14 ± 8.57	74	5	72	39	40	2–4			39	34	6	8
Ting Wang	2023	Meeting	Prospective	33	Tislelizumab	NA	NA	NA	NA	NA	NA	2			19	14		8
F. Cappuzzo	2024	Article	Retrospective	112	ICI	66 (41–84)	54	58	90	36	76	NA			80	27	6	8
J. Zhang	2024	Article	Retrospective	65	Tislelizumab	62.0 (57.0–66.0)	62	3	38	51	12	2–4			26	33	6	8
Bo Yang	2025	Article	Retrospective	10	Sintilimab	61.5 (54–72)	10	0	7	10	0	2–4			1	5	4	8
Mingliang Wang	2024	Meeting	Retrospective	113	ICI	NA	NA	NA	NA	NA	NA	2–4			NA			8

Immune checkpoint inhibitor (ICI) agents (Toripalimab, toripalimab; Tislelizumab, tislelizumab; Pembrolizumab, pembrolizumab; Sintilimab, sintilimab; Camrelizumab, camrelizumab; Durvalumab, durvalumab); Age, patient age [continuous variables presented as mean ± standard deviation or median (interquartile range)]; missing values labeled as NA; JACC, Journal of the American College of Cardiology Edition TNM staging.

### Quality assessment

The 34 studies were evaluated using the JBI Critical Appraisal Checklist for Case Series (48). Among the 34 included studies, the methodological quality was generally satisfactory. The majority of studies (*n* = 32, 94.1%) were assessed as having a low risk of bias, with “Yes” response proportions ranging from 73.0% to 100%. Three studies (*n* = 2, 5.9%) were categorized as having a medium risk of bias, with proportions between 50% and 61.8%. No study was judged to have a high risk of bias. The most common methodological limitations across studies were related to the unclear reporting of consecutive or complete participant inclusion (Q4 and Q5), insufficient detail in participant demographics or clinical information (Q6 and Q7), and, less frequently, inadequate reporting of follow-up outcomes (Q8) or statistical methods (Q10). The overall high proportion of low-risk studies supports the reliability of the synthesized evidence. The details of the quality assessment are presented in **Table 2**.

**Table 2:** Quality assessment of the studies included in the meta-analysis.doc.

Study	Year	Q1	Q2	Q3	Q4	Q5	Q6	Q7	Q8	Q9	Q10	Total
S. Yan	2021	Y	Y	Y	Y	Y	N	N	N	Y	N	6
T. Wang	2022	Y	Y	N	Y	Y	N	N	Y	Y	Y	7
Y. Zhang	2021	Y	Y	Y	N	Y	Y	N	Y	N	Y	7
X. Zhu	2021	Y	Y	Y	N	Y	N	N	N	Y	N	5
Hao Chen	2024	Y	Y	Y	Y	Y	N	N	N	Y	Y	7
Jing Zheng	2023	Y	Y	Y	Y	Y	Y	N	N	Y	Y	8
Hongyan Xu	2022	Y	Y	Y	Y	Y	N	N	N	Y	Y	7
Chao Sun	2024	Y	Y	Y	Y	Y	Y	Y	N	Y	Y	9
Y. Zhang	2022	Y	Y	Y	Y	Y	Y	N	N	Y	N	8
Y. Wang	2023	Y	Y	Y	Y	Y	N	N	N	N	N	5
Haoran Xia	2024	Y	Y	Y	Y	Y	Y	Y	N	Y	Y	9
X. Dong	2022	Y	Y	Y	Y	Y	Y	N	N	Y	N	8
Jing Guo	2023	Y	Y	Y	Y	Y	Y	Y	Y	N	Y	9
Tianxiang Chen	2021	Y	Y	Y	N	Y	Y	N	N	Y	Y	7
Jianzhen Shan	2024	Y	Y	Y	N	N	Y	Y	Y	N	Y	7
G. Jiang	2023	Y	Y	N	Y	Y	N	N	N	Y	N	5
K. Ma	2023	Y	Y	N	Y	Y	Y	Y	N	Y	N	7
Hongsheng Deng	2021	Y	Y	Y	Y	Y	Y	N	N	Y	Y	8
Ying Wang	2023	Y	Y	Y	Y	Y	Y	N	N	Y	Y	8
J.Z. Shan	2022	Y	Y	Y	Y	Y	N	N	N	Y	Y	7
Xuhua Huang	2024	Y	Y	Y	Y	Y	Y	Y	N	Y	Y	9
Min Tang	2024	Y	Y	Y	Y	Y	Y	Y	N	Y	Y	9
T. Wang	2024	Y	Y	Y	Y	Y	N	N	N	Y	Y	7
Biagio Ricciuti	2025	Y	Y	Y	Y	Y	Y	Y	N	Y	Y	9
Suyu Wang	2025	Y	Y	Y	Y	Y	Y	Y	N	Y	Y	9
Xiangyang Yu	2024	Y	Y	Y	Y	Y	Y	Y	N	Y	Y	9
Jianfeng Li	2024	Y	Y	Y	Y	Y	Y	Y	N	Y	Y	9
Yana Qi	2025	Y	Y	Y	Y	Y	Y	Y	N	Y	Y	9
Chengxiang Yi	2025	Y	Y	Y	Y	Y	Y	Y	N	Y	Y	9
Ting Wang	2023	Y	Y	Y	Y	Y	Y	Y	N	Y	Y	9
F. Cappuzzo	2024	Y	Y	Y	Y	Y	Y	Y	N	Y	Y	9
J. Zhang	2024	Y	Y	Y	Y	Y	N	N	N	Y	Y	7
Bo Yang	2025	Y	Y	Y	Y	Y	Y	N	N	Y	Y	8
Mingliang Wang	2024	Y	Y	Y	Y	Y	Y	Y	N	Y	Y	9

Numbers Q1–Q10 signify the following: Q1, were there clear criteria for inclusion in the case series? Q2, was the condition measured in a standard, reliable way for all participants included in the case series? Q3, were valid methods used for identification of the condition for all participants included in the case series? Q4, did the case series have consecutive inclusion of participants? Q5, did the case series have complete inclusion of participants? Q6, was there clear reporting of the demographics of the participants in the study? Q7, was there clear reporting of clinical information of the participants? Q8, were the outcomes or follow-up results of cases clearly reported? Q9, was there clear reporting of the presenting site(s)/clinic(s) demographic information? Q10, was statistical analysis appropriate? Total: Counts the total “Y” responses.

### Outcome measures

#### Surgical resection rates

**Figure 2:**
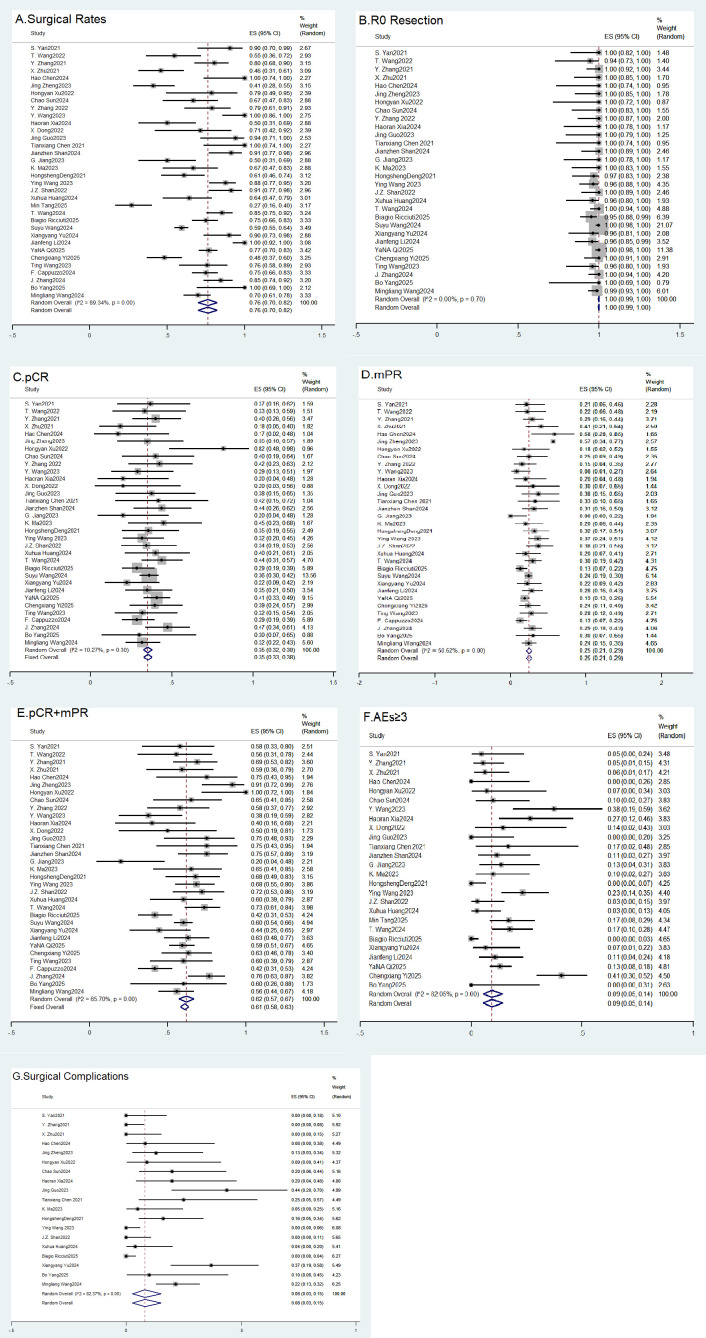
Forest plot of the square for each study indicates the point estimate, with its size proportional to the study weight. The horizontal line shows the 95% confidence interval. The diamond represents the pooled estimate calculated using a [fixed-effect/random-effects] model, with its width indicating the 95% confidence interval. Heterogeneity was quantified using the I² statistic.

All studies included in the analysis reported the primary outcome. The pooled surgical resection rate was 76.0% (95% CI: 70.0% to 82.0%, *I*^2^ = 89.34%, *p* < 0.001), as shown in **Figure 2A**. No significant publication bias was detected (Begg’s *p* = 0.342, Egger’s *p* = 0.064). The trim-and-fill method imputed 8 studies, adjusting the pooled rate to 66.4% (95% CI: 60.8% to 73.1%) (**Table 3**). Subgroup analysis based on study design revealed no statistically significant difference in the pooled surgical resection rates between prospective and retrospective studies (68% vs. 74%, between-subgroup *p* = 0.38), and this stratification did not reduce the observed heterogeneity. Meta-regression analysis further indicated no significant association between the effect size and its standard error (β = −2.22, *p* = 0.134), with the model demonstrating weak explanatory power for the heterogeneity (adjusted *R*^2^ = 5.96%). These analyses suggest that the observed substantial heterogeneity primarily stems from underlying clinical heterogeneity across studies, rather than methodological differences. This likely reflects variations in patient selection criteria, definitions of resectability, and institutional surgical practices.

**Table 3:** Combined results of publication bias tests, heterogeneity, and trim-and-fill analysis.

Indicator	Studies	Begg’s	Egger	*I* ^2^	Pre-trim-and-fill	Post-trim-and-fill
SC	19	0.07/0.08	0.29	82.37%	0.10 (0.02–0.18)	Consistent
Surgery rate	34	0.342/0.35	0.06	89.34%	0.692 (0.65–0.74)	0.66 (0.62–0.71)
R0 excision	30	0.02/0.02	0.67	0.00%	0.99 (0.94–1.04)	0.99 (0.94–1.04)
pCR + mPR	33	0.96/0.98	0.43	65.70%	0.60 (0.55–0.65)	0.58 (0.53–0.63)
pCR	34	0.62/0.64	0.86	13.94%	0.36 (0.31–0.41)	Consistent
mPR	33	0.70/0.71	0.14	50.62%	0.25 (0.19–0.30)	0.22 (0.17–0.27)
AES	26	0.83/0.84	0.59	82.05%	0.13 (0.07–0.18)	Consistent

#### R0 resection rates

A total of 30 studies were included. The pooled R0 resection rate was 100% (95% CI: 99% to 100%, *I*^2^ = 0.00%, *p* = 0.70), as shown in **Figure 2B**. It is crucial to provide context for this estimate: it applies only to the subset of patients who successfully completed neoadjuvant therapy and subsequently underwent resection. Patients with disease progression, severe toxicity, or those deemed inoperable upon re-evaluation were not included in this calculation. Therefore, this figure carries substantial selection bias and should not be interpreted as the probability of complete resection for all enrolled participants (i.e., the intention-to-treat population). Additionally, in retrospective studies, patients who did not undergo surgery may have been excluded from the analysis. The fixed-effects model was used due to negligible heterogeneity. Sensitivity analysis confirmed stability (**Supplementary Figure S2B**). Egger’s test showed no significant bias (*p* = 0.666). The trim-and-fill method imputed 1 study, with the adjusted rate remaining at 98.8% (95% CI: 93.5% to 104.2%) (**Table 3**).

### pCR

All studies included in the analysis reported the pCR of neoadjuvant immunochemotherapy in patients with potentially resectable NSCLC. The pooled pCR rate was 35.9% (95% CI: 30.7% to 41.0%, *I*^2^ = 13.94%, *p* = 0.24), as shown in **Figure 2C**. The fixed-effects model was used due to low heterogeneity. Sensitivity analysis confirmed the robustness of the results (**Supplementary Figure S2C**). Begg’s test (*p* = 0.624) and Egger’s test (*p* = 0.862) indicated no significant publication bias. The trim-and-fill analysis did not impute any missing studies (**Table 3**).

### mPR

A total of 33 studies were included. The pooled proportion (metaprop) of the outcome (mPR) following surgery was 0.25 (95% CI: 0.21–0.29), as shown in **Figure 2D**. The random-effects model was applied. Sensitivity analysis showed stable results (**Supplementary Figure S2D**). No significant publication bias was detected (Begg’s *p* = 0.698, Egger’s *p* = 0.144). The trim-and-fill method imputed seven studies, adjusting the pooled rate to 22.2% (95% CI: 17.4% to 27.1%) (**Table 3**).

### pCR + mPR

A total of 33 studies reported data for this composite endpoint. The pooled pCR + mPR rate was 60.2% (95% CI: 55.1% to 65.4%, *I*^2^ = 65.70%, *p* < 0.001), as shown in **Figure 2E**. The random-effects model was used. Sensitivity analysis confirmed stability (**Supplementary Figure S2E**). No significant publication bias was found (Begg’s *p* = 0.963, Egger’s *p* = 0.433). The trim-and-fill method imputed five studies, adjusting the pooled rate to 58.4% (95% CI: 53.4% to 63.3%) (**Table 3**).

### AEs

A total of 26 studies were included. The pooled rate of grade ≥3 AEs was 9.0% (95% CI: 5.0% to 14.0%, *I*^2^ = 82.05%, *p* < 0.001), as shown in **Figure 2F**. Sensitivity analysis confirmed robustness (**Supplementary Figure S2F**). Trim-and-fill analysis revealed that no trimming was needed. No significant publication bias was detected (Begg’s/Egger’s tests) (**Table 3**). Subgroup analysis by study design revealed no significant difference in pooled incidence rates between prospective and retrospective studies (13% vs. 12%, between-subgroup *p* = 0.79), indicating that this factor only partially explains the heterogeneity. Meta-regression analysis demonstrated a significant positive correlation between the effect size and its standard error (β = 2.55, *p* = 0.014), suggesting that study precision is an important source of heterogeneity. Studies with less precise estimates may have over-reported incidence rates. This model explained 38.5% of the between-study variance, indicating variations in the reporting, grading, or management of AEs across different studies.

### Surgical complications

A total of 19 studies were included. The pooled SC rate was 8.0% (95% CI: 3.0% to 15.0%, *I*^2^ = 82.37%, *p* < 0.001), as shown in **Figure 2G**. Funnel plots and Egger’s test (bias = 0.547, *p* = 0.290) and Begg’s test (*p* = 0.074–0.080) suggested no strong evidence of publication bias. Trim-and-fill analysis did not require trimming (no asymmetry detected). Meta-regression did not identify a significant covariate effect (**Table 3**). To explore the sources of heterogeneity, we first conducted a subgroup analysis based on study design. The pooled complication rates were 19% for prospective studies and 15% for retrospective studies, with no statistically significant difference between the subgroups (*p* = 0.57), indicating that study design type was not a major source of heterogeneity. Subsequently, we employed meta-regression analysis for further exploration and found a significant positive correlation between the standard error of the effect size and its magnitude (β = 2.42, *p* = 0.038). This model explained approximately 43.6% of the between-study variance. This suggests that the estimated complication rates in the studies are related to their estimation precision: studies reporting higher incidence rates exhibited greater uncertainty. Therefore, the observed heterogeneity likely stems primarily from differences in sample sizes and variability of effect sizes across studies, rather than from methodological differences in study design.

### Sensitivity analyses

To assess the potential bias introduced by including conference abstracts, a sensitivity analysis was performed by excluding all studies identified as conference abstracts. The recalculated pooled estimates for major outcomes (surgical resection rate, pCR, mPR, and grade ≥3 AEs) showed no substantial difference from the primary analysis including all studies (detailed results are presented in **Supplementary Figure S4**). This suggests that the inclusion of conference abstracts did not materially alter the overall conclusions of this meta-analysis.

### Inter-study heterogeneity and bias assessment

Funnel plots were created for each outcome measure to detect publication bias in the meta-analysis (**Supplementary Figure S3**). A leave-one-out sensitivity analysis was performed, and no single study significantly altered the overall result (**Supplementary Figure S2**). Egger’s and Begg’s test results are shown in **Table 3**. The high degree of heterogeneity (*I*^2^ > 80%) observed for outcomes such as surgical resection rate, grade ≥3 AEs, and surgical complication rate warrants careful interpretation. This heterogeneity likely stems from multiple factors, including differences in patient selection criteria, definitions of resectability across centers, surgical expertise and volume, and variability in the reporting or grading of AEs. Consequently, the pooled estimates for these outcomes should be viewed with increased uncertainty.

## Discussion

This study evaluated the feasibility of neoadjuvant immunochemotherapy in patients with potentially resectable NSCLC. A meta-analysis of 34 studies (1,885 patients) suggests that potential feasibility of neoadjuvant immunochemotherapy followed by surgical resection was feasible for this cohort.

The meta-analysis revealed that 76% of patients achieved R0 resection (95% CI: 0.70–0.82), consistent with historical data reporting resection rates of (83.2%–93.0%) (7, 8) in patients with resectable NSCLC. The pCR rate in patients receiving neoadjuvant immunochemotherapy was 35% (95% CI: 0.32–0.38), compared to 26.31% in patients with resectable NSCLC (49). The combined rate of pCR + mPR was 62% (95% CI: 0.57–0.67) (50). The pooled incidence of grade ≥3 AEs in our analysis was 9% (95% CI: 0.05–0.14). It is crucial to interpret this figure with caution when comparing it to rates reported in pivotal trials like CheckMate 816 (33.5%). Direct comparisons are limited by differences in patient populations (potentially resectable vs. resectable), treatment protocols, study designs, and, likely, the intensity of AE monitoring and reporting (10). Post-neoadjuvant surgery-related complications occurred in 8% (95% CI: 0.03–0.15), lower than the 11.4% reported in prior studies. These findings suggest substantial pathological responses in patients with potentially resectable NSCLC. The results provide supportive evidence for the activity of neoadjuvant immunochemotherapy in this population (50–53). Safety profiles were favorable, with no new safety signals compared to surgery-only cohorts.

While neoadjuvant immunochemotherapy significantly enhances tumor pathological response, the local inflammatory reaction and tissue repair processes it induces concomitantly substantially increase the technical complexity of thoracic surgery and the difficulty of perioperative decision-making (54, 55). The core paradox lies in the fact that treatment-induced extensive fibrosis and dense adhesions obscure critical anatomical planes (e.g., the pulmonary hilum and mediastinum). This not only renders vascular dissection and lymph node dissection (particularly in cases forming “stationary lymph nodes”) exceedingly challenging, thereby increasing the risk of vascular injury and hemorrhage, but also may compel the surgeon to extend the resection margin or convert to a thoracotomy (56). Although large phase III clinical trials conducted within the context of strictly selected expert centers have reported manageable rates of surgical complications, these findings may not fully represent the challenges encountered in broader clinical practice. This is because a profound pathological response may coexist with more severe fibrosis, and the surgeon’s subjective experience of technical difficulty is often not fully captured by objective metrics such as “operative time” or “blood loss.” Consequently, future clinical practice necessitates the integration of more refined preoperative assessments (e.g., radiomics) to predict surgical risk. Furthermore, it mandates that such procedures be performed in high-volume, experienced centers following careful evaluation by an MDT, aiming to strike an optimal balance between pursuing oncological radicality and ensuring procedural safety along with patient quality of life.

While this study highlights promising outcomes, its optimistic results may reflect selection biases. Included studies predominantly enrolled smokers (57), patients with squamous cell carcinoma (58), or those from high-volume centers, potentially overestimating response rates due to higher pathologic remission rates in these subgroups. Retrospective designs and reliance on historical controls may introduce confounding, such as excluding patients with progressive disease post-neoadjuvant therapy. Additionally, the emphasis on pCR/mPR as primary endpoints may overlook the benefits of radical resection. As demonstrated by the CheckMate 816 trial (59), a survival advantage for surgical resection compared to the non-surgical group exists even in the absence of pCR/mPR.

For patients with locally advanced NSCLC that preclude direct surgical resection, the current standard therapeutic regimen is the PACIFIC paradigm, which entails concurrent chemoradiotherapy followed by consolidation immunotherapy. Neoadjuvant immunochemotherapy offers a potential pathway to radical surgery for initially inoperable NSCLC. This study provides novel evidence supporting its use in potentially resectable cases, warranting further validation in clinical trials.

### Limitations

This meta-analysis has several important limitations that warrant consideration when interpreting the findings. First, the predominance of single-arm trials and small cohort studies—without inclusion of randomized controlled trials (RCTs) or phase III trials—introduces potential biases in key outcome measures such as surgical complication rates, R0 resection rates, and surgical resectability metrics. The lack of comparative data from RCTs makes it difficult to draw definitive conclusions about the relative feasibility of neoadjuvant immunochemotherapy compared to standard treatments like the PACIFIC regimen (concurrent chemoradiotherapy followed by durvalumab maintenance).

Second, the immature long-term survival data currently available preclude robust evaluation of whether the observed improvements in pathological responses (pCR/mPR) translate into meaningful PFS or OS benefits. While pathological response rates are promising surrogate endpoints, their correlation with long-term survival outcomes remains to be fully validated in this patient population.

Third, the complexity of surgical interventions following neoadjuvant immunochemotherapy may elevate perioperative risks, necessitating involvement of highly experienced thoracic surgeons. The observed surgical complication rate of 6% (95% CI: 0.03–0.09) may underestimate real-world risks, as the included studies were predominantly conducted at high-volume centers with specialized surgical expertise.

Fourth, the definition of “potentially resectable” status exhibits subjectivity dependent on MDT assessment and institutional practices. The inclusion of heterogeneous patient populations—particularly those with N2/N3 lymph node involvement or T3–4 tumors—raises unresolved questions about the generalizability of R0 resection rates and survival outcomes across different clinical scenarios.

To mitigate these concerns, we implemented rigorous statistical methods including subgroup analyses and publication bias correction (Egger’s test + trim-and-fill method). However, the substantial between-study heterogeneity (*I*^2^ > 50% for most outcomes) and small sample sizes in many included studies (total *n* = 1,885) warrant cautious interpretation of the pooled estimates. The trim-and-fill analysis revealed significant publication bias for R0 resection rates and surgical complications, suggesting that the reported effect sizes may overestimate true treatment effects. These limitations highlight the need for larger, RCTs with standardized definitions of resectability and longer follow-up to validate these preliminary findings.

## Conclusion

In summary, this single-arm meta-analysis indicates that for patients with potentially resectable NSCLC as assessed by MDT, neoadjuvant immunochemotherapy followed by surgery is associated with considerable short-term pathological response rates (pCR 35.9%, pCR + mPR 60.2%), an achievable surgical resection rate (76.0%), and seemingly manageable rates of grade ≥3 treatment-related AEs and surgical complications. However, because of the inherent limitations of single-arm designs, clinical heterogeneity, potential selection biases, and the absence of mature survival data, these findings must be considered preliminary and exploratory. They do not establish NICT as a standard of care for this population, nor do they allow for direct efficacy comparisons with the PACIFIC regimen. The primary value of this study lies in providing preliminary evidence for exploring a novel strategy aimed at achieving radical surgery through downstaging, as an alternative to the current standard of chemoradiation followed by immunotherapy. Therefore, we strongly advocate for and highlight the urgent need for well-designed, adequately powered randomized phase III trials. Such trials should directly compare the efficacy and safety of neoadjuvant immunochemotherapy followed by surgery versus the current standard therapy (e.g., the PACIFIC regimen) in patients with potentially resectable NSCLC, to definitively determine its clinical value and optimal place in the treatment paradigm.

## Data Availability

The original contributions presented in the study are included in the article/**Supplementary Material**. Further inquiries can be directed to the corresponding authors.

## Supplementary material

Supplementary Figure S1 Sensitivity analysis of the pooled results.

Supplementary Figure S2 Funnel plot of the pooled results.

Supplementary Figure S3 Subgroup analysis by study design.

Supplementary Figure S4 Forest plot for subgroup.

Supplementary Table S1 Database retrieval strategy.
