# Supplementary figures and images for: Feasibility of neoadjuvant immunochemotherapy in potentially resectable non-small cell lung cancer: a single-arm meta-analysis
Source: Front Oncol. 2026 May 7;16:1826159. doi: 10.3389/fonc.2026.1826159 (PMC13189883; doi:10.3389/fonc.2026.1826159)

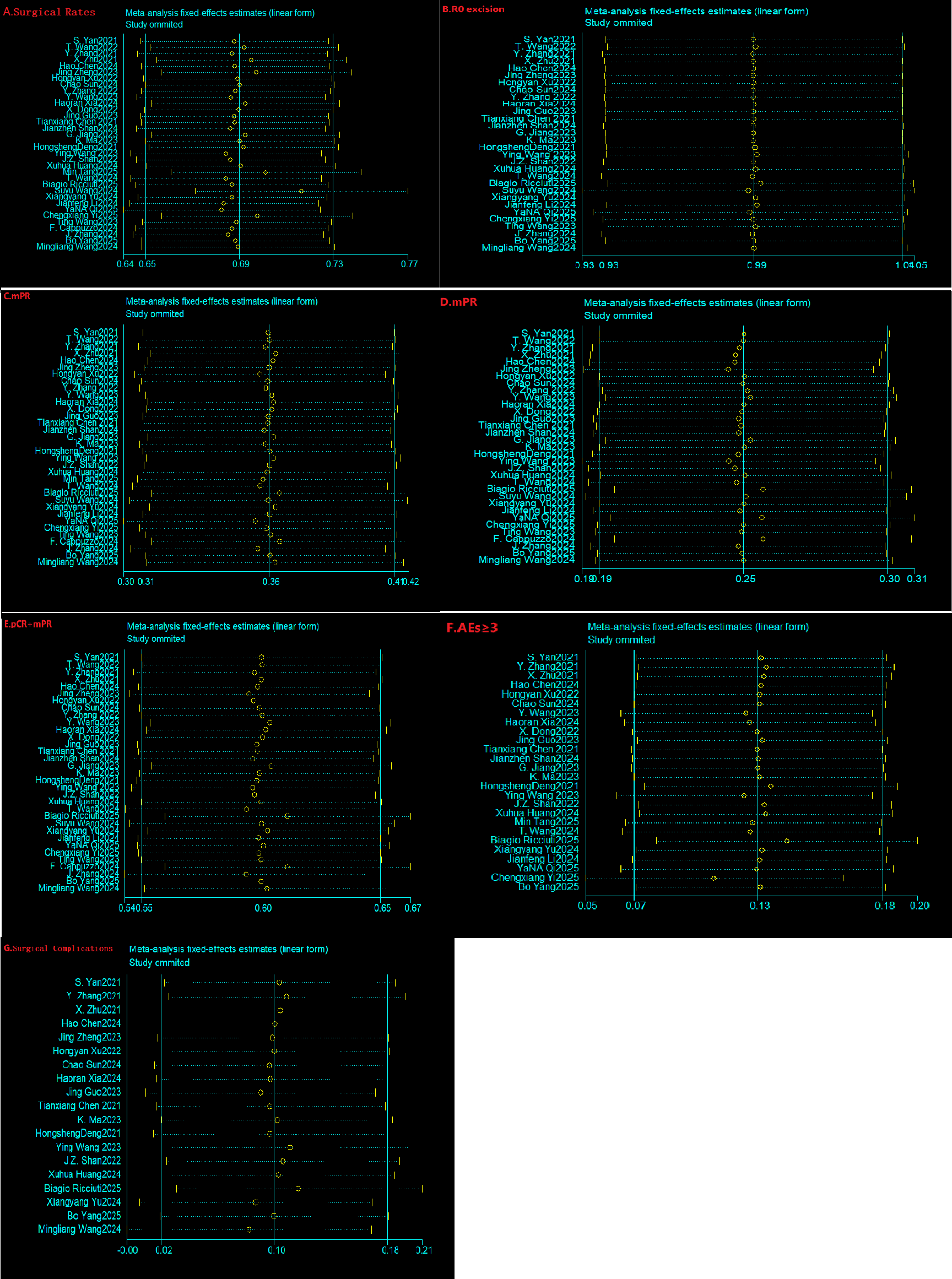

Supplement: Supplementary Figure S1 — Sensitivity analysis of the pooled results. [file Image1.tif]

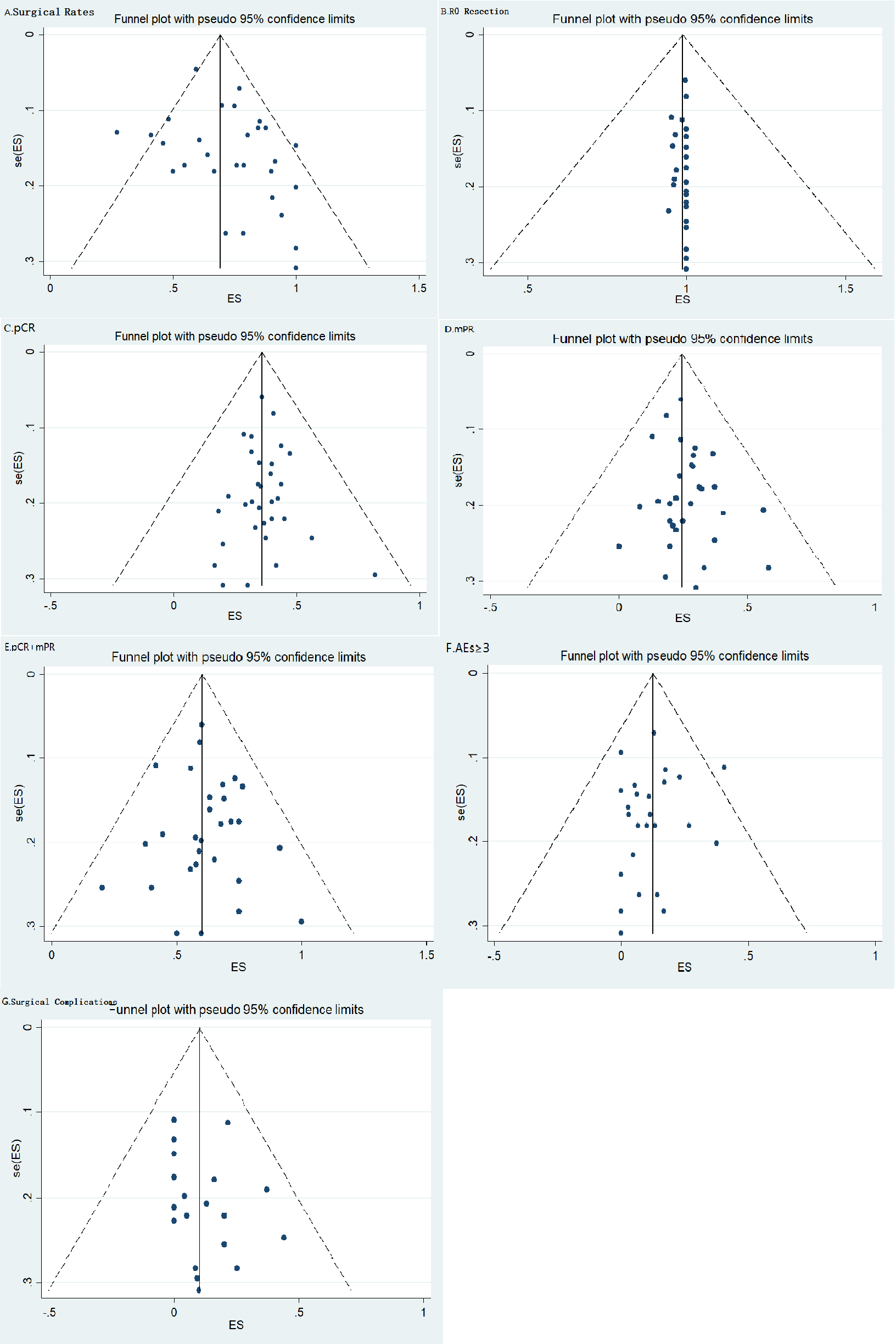

Supplement: Supplementary Figure S2 — Funnel plot of the pooled results. [file Image2.tif]

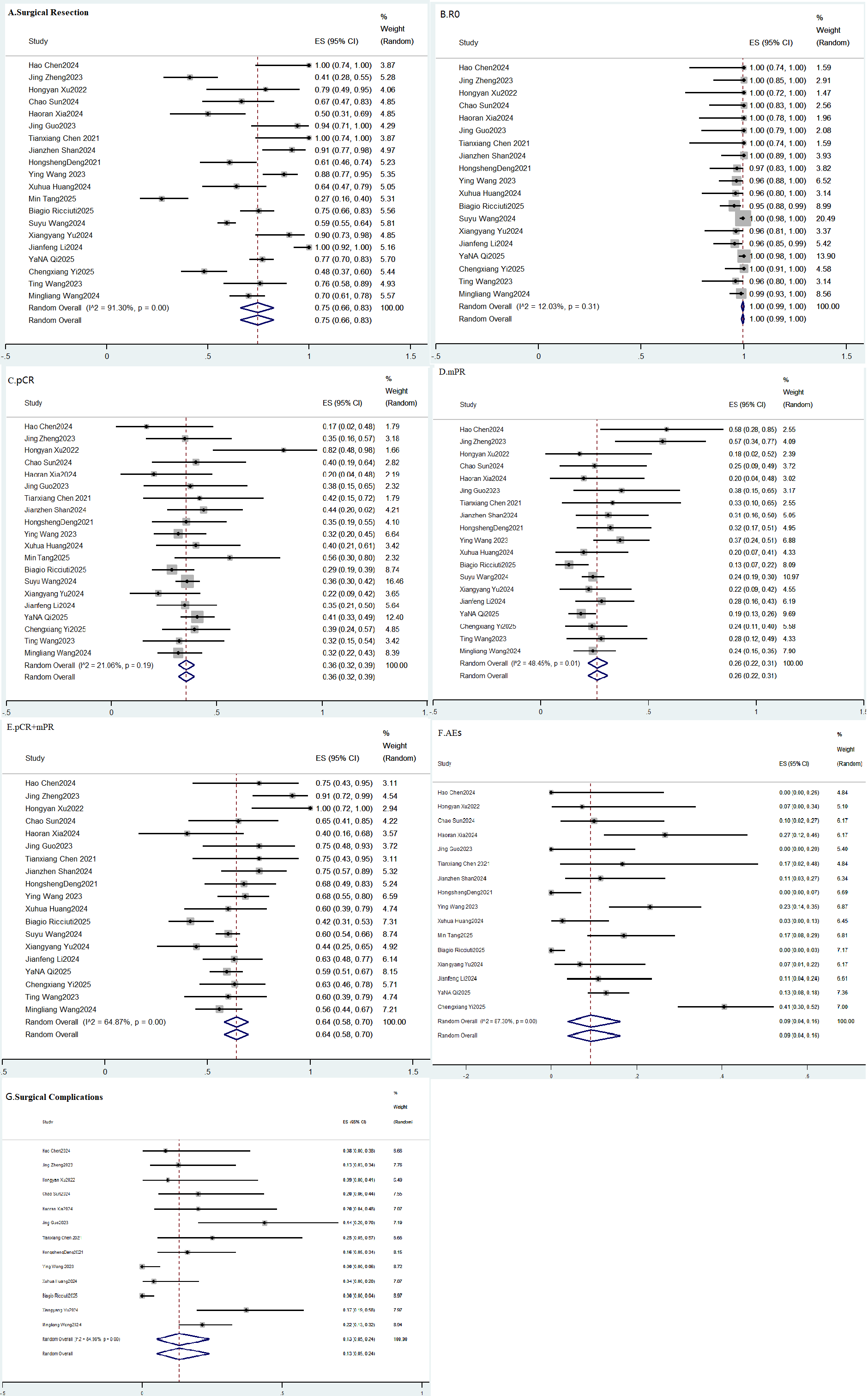

Supplement: Supplementary Figure S3 — Subgroup analysis by study design. [file Image3.tif]

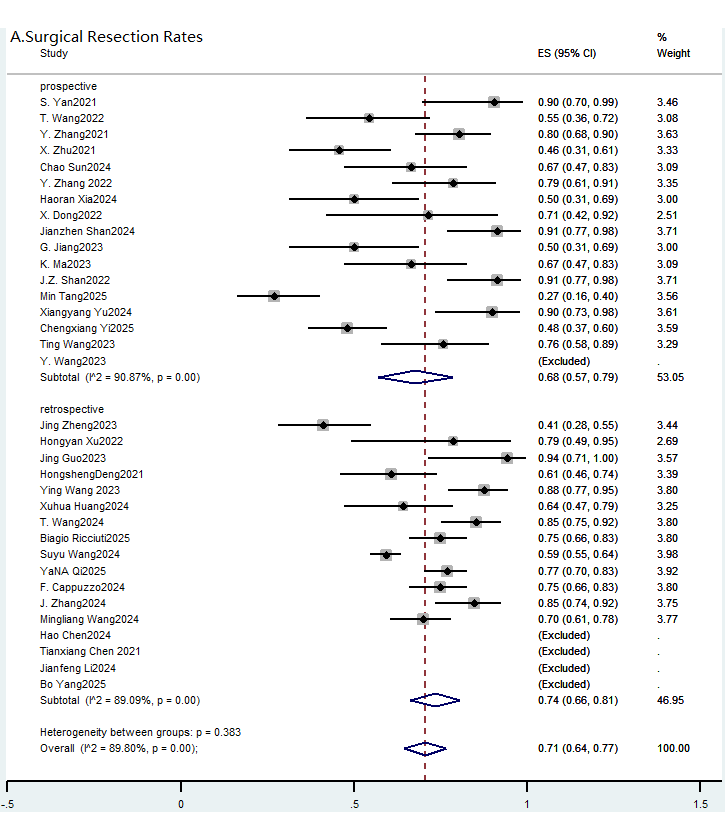


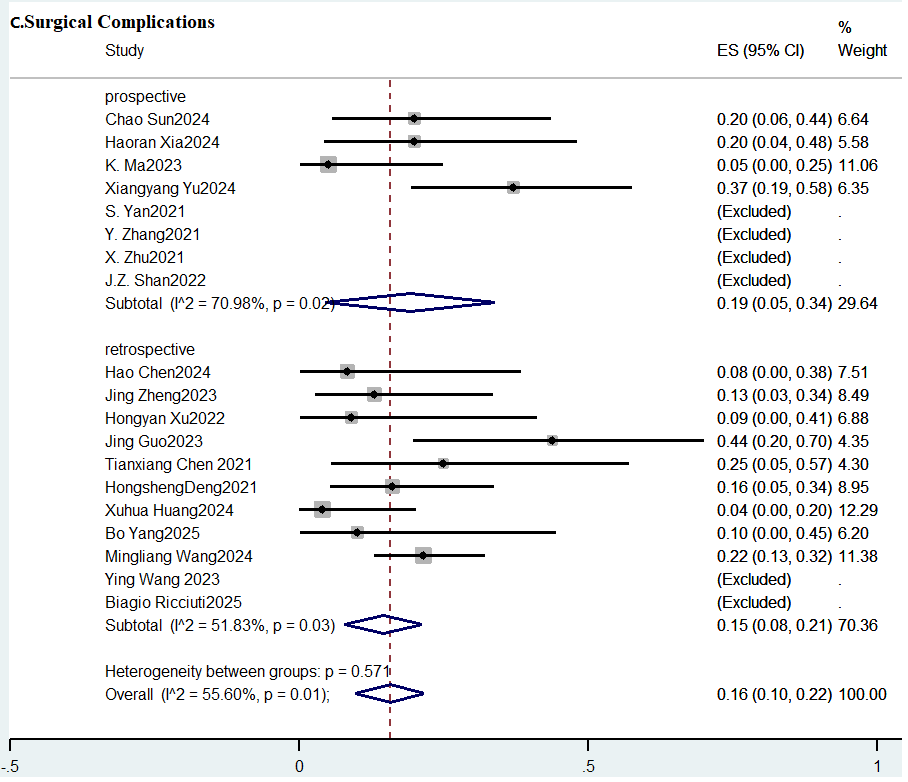


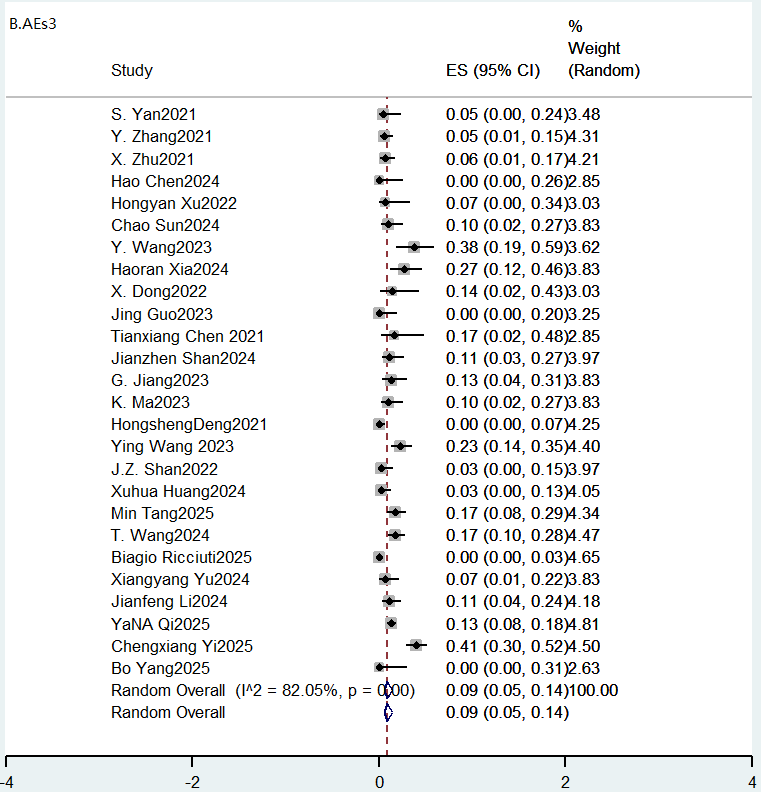

Supplement: Supplementary Table S1 — Database retrieval strategy. [file Table1.docx]
