# Supplementary material for: Feasibility of neoadjuvant immunochemotherapy in potentially resectable non-small cell lung cancer: a single-arm meta-analysis
Source: Front Oncol. 2026 May 7;16:1826159. doi: 10.3389/fonc.2026.1826159 (PMC13189883; doi:10.3389/fonc.2026.1826159)
Supplement: Supplementary Figure S4 — Forest plot for subgroup. [file DataSheet1.doc]

| PUBMED | ((Potentially resectable[Title/Abstract] OR(Unresectable[Title/Abstract]) OR(Locally advanced[Title/Abstract]) OR(III stage[Title/Abstract])) AND ((Carcinoma, Non Small Cell Lung[Title/Abstract]) OR (Carcinomas, Non-Small-Cell Lung[Title/Abstract]) OR (Lung Carcinoma, Non-Small-Cell[Title/Abstract]) OR (Lung Carcinomas, Non-Small-Cell[Title/Abstract]) OR (Non-Small-Cell Lung Carcinomas[Title/Abstract]) OR (Carcinoma, Non-Small Cell Lung[Title/Abstract]) OR (Non-Small Cell Lung Cancer[Title/Abstract]) OR (Non-Small-Cell Lung Carcinoma[Title/Abstract]) OR (Non Small Cell Lung Carcinoma[Title/Abstract]) OR (Nonsmall Cell Lung Cancer[Title/Abstract]))) AND ((Neoadjuvant Therapies[Title/Abstract]) OR (Therapy, Neoadjuvant[Title/Abstract]) OR (Neoadjuvant Treatment[Title/Abstract]) OR (Neoadjuvant Treatments[Title/Abstract]) OR (Treatment, Neoadjuvant[Title/Abstract]) OR (Neoadjuvant Chemotherapy[Title/Abstract]) OR (Chemotherapy, Neoadjuvant[Title/Abstract]) OR (Neoadjuvant Chemotherapies[Title/Abstract]) OR (Neoadjuvant Chemotherapy Treatment[Title/Abstract]) OR (Chemotherapy Treatment, Neoadjuvant[Title/Abstract]) OR (Neoadjuvant Chemotherapy Treatments[Title/Abstract]) OR (Treatment, Neoadjuvant Chemotherapy[Title/Abstract]) OR (Neoadjuvant Systemic Therapy[Title/Abstract]) OR (Neoadjuvant Systemic Therapies[Title/Abstract]) OR (Systemic Therapy, Neoadjuvant[Title/Abstract]) OR (Therapy, Neoadjuvant Systemic[Title/Abstract]) OR (Neoadjuvant Systemic Treatment[Title/Abstract]) OR (Neoadjuvant Systemic Treatments[Title/Abstract]) OR (Systemic Treatment, Neoadjuvant[Title/Abstract]) OR (Treatment, Neoadjuvant Systemic[Title/Abstract])) |
| --- | --- |
| EMBASE | Query  #1 AND #3 AND #5  #2 OR #4  'chemo-immunotherapy' OR 'immuno-chemotherapy' OR 'immunochemotherapy' OR 'chemoimmunotherapy' OR 'chemoimmunotherapy'/exp  'potentially resectable':ab,ti OR 'unresectable':ab,ti OR 'locally advanced':ab,ti OR 'iii stage':ab,ti  'neo-adjuvant therapy' OR 'neo-adjuvant treatment' OR 'neoadjuvant treatment' OR 'neoadjuvant therapy' OR 'neoadjuvant therapy'/exp  'bronchial non small cell cancer' OR 'bronchial non small cell carcinoma' OR 'carcinoma, non-small-cell lung' OR 'lung cancer, non small cell' OR 'lung non small cell cancer' OR 'lung non small cell carcinoma' OR 'non oat cell lung cancer' OR 'non small cell bronchial cancer' OR 'non small cell cancer, lung' OR 'non small cell lung carcinoma' OR 'non small cell pulmonary cancer' OR 'non small cell pulmonary carcinoma' OR 'non squamous nsclc' OR 'non-oat cell lung cancer' OR 'non-small-cell lung carcinoma' OR 'nonsmall cell carcinoma of the lung' OR 'nonsmall cell lung cancer' OR 'nonsmall cell lung carcinoma' OR 'pulmonary non small cell cancer' OR 'pulmonary non small cell carcinoma' OR 'non small cell lung cancer' OR 'non small cell lung cancer'/exp |
| Cochrane | ID Search Hits  #1 MeSH descriptor: [Carcinoma, Non-Small-Cell Lung] explode all trees  #2 (Lung Carcinomas, Non-Small-Cell):ti,ab,kw OR ( Non-Small-Cell Lung Carcinomas):ti,ab,kw OR ( Non-Small-Cell Lung Carcinoma):ti,ab,kw OR ( Carcinomas, Non-Small-Cell Lung):ti,ab,kw OR ( Non-Small Cell Lung Cancer):ti,ab,kw OR ( Carcinoma, Non-Small Cell Lung):ti,ab,kw OR ( Lung Carcinoma, Non-Small-Cell):ti,ab,kw OR ( Carcinoma, Non Small Cell Lung):ti,ab,kw OR ( Non-Small Cell Lung Carcinoma):ti,ab,kw OR ( Non Small Cell Lung Carcinoma):ti,ab,kw OR ( Nonsmall Cell Lung Cancer):ti,ab,kw  #3 (Potentially resectable):ti,ab,kw OR (Unresectable):ti,ab,kw OR (Locally advanced):ti,ab,kw OR (III stage):ti,ab,kw  #4 MeSH descriptor: [Chemoradiotherapy] explode all trees  #5 (Concurrent Radiochemotherapies):ti,ab,kw OR ( Concomitant Radiochemotherapy):ti,ab,kw OR ( Radiochemotherapy, Concomitant):ti,ab,kw OR ( Synchronous Chemoradiotherapies):ti,ab,kw OR ( Concurrent Chemoradiotherapy):ti,ab,kw OR ( Radiochemotherapy, Concurrent):ti,ab,kw OR ( Radiochemotherapies, Concurrent):ti,ab,kw OR ( Chemoradiotherapy, Synchronous):ti,ab,kw OR ( Concurrent Chemoradiotherapies):ti,ab,kw OR ( Chemoradiotherapy, Concomitant):ti,ab,kw OR ( Concomitant Chemoradiotherapy):ti,ab,kw OR ( Chemoradiotherapy, Concurrent):ti,ab,kw OR ( Concomitant Radiochemotherapies):ti,ab,kw OR ( Radiochemotherapies, Concomitant):ti,ab,kw OR ( Chemoradiotherapies, Concurrent):ti,ab,kw OR ( Chemoradiotherapies, Concomitant):ti,ab,kw OR ( Synchronous Chemoradiotherapy):ti,ab,kw OR ( Concomitant Chemoradiotherapies):ti,ab,kw OR ( Concurrent Radiochemotherapy):ti,ab,kw OR ( Chemoradiotherapies, Synchronous):ti,ab,kw OR ( Chemoradiotherapies):ti,ab,kw OR ( Radiochemotherapy):ti,ab,kw OR ( Radiochemotherapies):ti,ab,kw  #6 MeSH descriptor: [Immune Checkpoint Inhibitors] explode all trees  #7 (Checkpoint Blockade, Immune):ti,ab,kw OR (Immune Checkpoint Inhibition):ti,ab,kw OR (Immune Checkpoint Blockade):ti,ab,kw OR (Checkpoint Inhibition, Immune):ti,ab,kw OR (Programmed Cell Death Protein 1 Inhibitor):ti,ab,kw OR (PD-1 Inhibitor):ti,ab,kw OR (PD 1 Inhibitor):ti,ab,kw OR (Programmed Cell Death Protein 1 Inhibitors):ti,ab,kw OR (PD-1 Inhibitors):ti,ab,kw OR (Inhibitor, PD-1):ti,ab,kw OR (PD 1 Inhibitors):ti,ab,kw OR (Immune Checkpoint Blockers):ti,ab,kw OR (Checkpoint Inhibitors, Immune):ti,ab,kw OR (Checkpoint Blockers, Immune):ti,ab,kw OR (Checkpoint Inhibitor, Immune):ti,ab,kw OR (Immune Checkpoint Inhibitor):ti,ab,kw  #8 MeSH descriptor: [Neoadjuvant Therapy] explode all trees  #9 (Chemotherapy Treatment, Neoadjuvant):ti,ab,kw OR ( Chemotherapy, Neoadjuvant):ti,ab,kw OR ( Neoadjuvant Chemotherapy):ti,ab,kw OR ( Neoadjuvant Chemotherapies):ti,ab,kw OR ( Neoadjuvant Chemotherapy Treatments):ti,ab,kw OR ( Neoadjuvant Chemotherapy Treatment):ti,ab,kw OR ( Treatment, Neoadjuvant Chemotherapy):ti,ab,kw OR ( Treatment, Neoadjuvant Chemoradiation):ti,ab,kw OR ( Neoadjuvant Chemoradiation Therapies):ti,ab,kw OR ( Neoadjuvant Chemoradiation Treatment):ti,ab,kw OR ( Neoadjuvant Chemoradiotherapy):ti,ab,kw OR ( Chemoradiation Treatment, Neoadjuvant):ti,ab,kw OR ( Neoadjuvant Chemoradiations):ti,ab,kw OR ( Neoadjuvant Chemoradiation):ti,ab,kw OR ( Neoadjuvant Chemoradiation Therapy):ti,ab,kw OR ( Neoadjuvant Chemoradiotherapies):ti,ab,kw OR ( Therapy, Neoadjuvant Chemoradiation):ti,ab,kw OR ( Chemoradiation, Neoadjuvant):ti,ab,kw OR ( Chemoradiotherapy, Neoadjuvant):ti,ab,kw OR ( Neoadjuvant Chemoradiation Treatments):ti,ab,kw OR ( Chemoradiation Therapy, Neoadjuvant):ti,ab,kw OR ( Neoadjuvant Systemic Treatments):ti,ab,kw OR ( Neoadjuvant Systemic Treatment):ti,ab,kw OR ( Therapy, Neoadjuvant Systemic):ti,ab,kw OR ( Treatment, Neoadjuvant Systemic):ti,ab,kw OR ( Systemic Treatment, Neoadjuvant):ti,ab,kw OR ( Systemic Therapy, Neoadjuvant):ti,ab,kw OR ( Neoadjuvant Systemic Therapy):ti,ab,kw OR ( Neoadjuvant Systemic Therapies):ti,ab,kw OR ( Radiation, Neoadjuvant):ti,ab,kw OR ( Therapy, Neoadjuvant Radiation):ti,ab,kw OR ( Treatment, Neoadjuvant Radiation):ti,ab,kw OR ( Neoadjuvant Radiations):ti,ab,kw OR ( Radiotherapy, Neoadjuvant):ti,ab,kw OR ( Radiation Therapy, Neoadjuvant):ti,ab,kw OR ( Neoadjuvant Radiation Therapy):ti,ab,kw OR ( Radiation Treatment, Neoadjuvant):ti,ab,kw OR ( Neoadjuvant Radiation):ti,ab,kw OR ( Neoadjuvant Radiotherapies):ti,ab,kw OR ( Neoadjuvant Radiation Therapies):ti,ab,kw OR ( Neoadjuvant Radiation Treatment):ti,ab,kw OR ( Neoadjuvant Radiotherapy):ti,ab,kw OR ( Neoadjuvant Radiation Treatments):ti,ab,kw OR ( Treatment, Neoadjuvant):ti,ab,kw OR ( Therapy, Neoadjuvant):ti,ab,kw OR ( Neoadjuvant Treatment):ti,ab,kw OR ( Neoadjuvant Treatments):ti,ab,kw OR ( Neoadjuvant Therapies):ti,ab,kw  #10 #1 or #2  #11 #5 or #4  #12 #6 or #7  #13 #8 or #9  #14 #3 and #10  #15 #11 or #13  #16 #15 and #12 and #14 |
